# The Evolution of Novelty in Conserved Gene Families

**DOI:** 10.1155/2012/490894

**Published:** 2012-06-19

**Authors:** Gabriel V. Markov, Ralf J. Sommer

**Affiliations:** Department for Evolutionary Biology, Max Planck Institute for Developmental Biology, Spemannstraße 37, 72076 Tübingen, Germany

## Abstract

One of the major aims of contemporary evolutionary biology is the understanding of the current pattern of biological diversity. This involves, first, the description of character distribution at various nodes of the phylogenetic tree of life and, second, the functional explanation of such changes. The analysis of character distribution is a powerful tool at both the morphological and molecular levels. Recent high-throughput sequencing approaches provide new opportunities to study the genetic architecture of organisms at the genome-wide level. In eukaryotes, one overarching finding is the absence of simple correlations of gene count and biological complexity. Instead, the domain architecture of proteins is becoming a central focus for large-scale evolutionary innovations. Here, we review examples of the evolution of novelty in conserved gene families in insects and nematodes. We highlight how in the absence of whole-genome duplications molecular novelty can arise, how members of gene families have diversified at distinct mechanistic levels, and how gene expression can be maintained in the context of multiple innovations in regulatory mechanisms.

## 1. Introduction

 To understand evolutionary novelty and its contribution to the generation of new species, biologists search for character differences between closely related species and try to determine the functional meaning of such changes. Characters range from morphological traits, like the trichome pattern on the cuticle of a fruit fly larva, to molecular characters such as nucleotide sequences. The genomics era is now providing an increasing number and also new kinds of molecular characters, such as gene numbers in multigenic families, gene position on chromosomes, microRNAs, or insertions of mobile elements at various places in a genome. In addition, next-generation sequencing approaches provide genome-wide single-nucleotide polymorphism (SNP) and copy number variation (CNV) data in a number of model organisms [1]. Nonetheless, it remains challenging to articulate knowledge concerning all these characters in a comprehensive functional framework. While there are some cases with a direct link between various levels of character changes, such as the small number of single-nucleotide mutations in a transcriptional enhancer that can modify the trichome pattern on the larval cuticle in *Drosophila sechellia* [2], there are many other cases where great variation at the molecular level has no simply interpretable effect at the level of the organism. Although this does not mean that such changes are necessarily neutral, there is a strong tendency to correlate any unexpected genomic finding, such as genome duplication or peculiar gene family expansion, with an adaptation to special environmental conditions, often without proper justification [3]. These attempts reflect an understandable quest for generalization. It was recently stressed that evolutionary relevant mutations are not distributed at random in the genome, and that a precise understanding of the contribution of genetic factors to evolution requires the consideration of the specific functional properties of genes [4]. One aspect that is often forgotten in these discussions is that cross-genome comparisons between species are mostly challenged by the inherent difficulty to infer homology between deeply rooted species [3–5].

 Here, we review some cases where spectacular molecular changes do not correlate with any clear phenotypic novelty at the organismal level, and we highlight the need to cope with different types of variation to understand their reciprocal interactions. We will review three examples dealing with (i) novelty by genome diversification in the absence of whole-genome duplication, (ii) novelty in large gene families, and (iii) novelty in promoter regions. For practical reasons, we focus our example choices on ecdysozoans, an animal group that contains two of the best genetic models, the fruit fly *Drosophila melanogaster* and the nematode worm *Caenorhabditis elegans*, each of which is complemented by satellite organisms, allowing us to make sophisticated comparisons by functional investigations [6].

## 2. Genome Diversification in the Absence of Whole-Genome Duplication

 The spectacular examples of land plants and vertebrates highlight the importance of genome duplications for evolutionary success measured in a number of ways, such as number of species, morphological innovations, and ecological diversification [7]. In the animals, however, two other phyla outcompete the vertebrates in all these characteristics. Insects and nematodes are the largest animal phyla with respect to species number as well as morphological and ecological diversification [8, 9]. It is often forgotten that they managed to reach the highest levels of species diversity among animals without the involvement of genome duplication. At the same time, it has to be stressed that the absence of genome duplications in insects and nematodes does not mean that these two groups are lacking noticeable genomic innovations. For example, genome-wide comparisons of aminoacid substitution patterns lead to the estimate that the 39-million-year time interval between the separation of dipterans and coleopterans and the split of the two main dipteran lineages was characterized by an episodic threefold increase in evolutionary rate relative to the mean rate found for the coleopteran representative *Tribolium castaneum* [10]. It was then established that lepidopterans have branches of similar length than dipterans, whereas other holometabolous insects have shorter branches, indicating substitution rates comparable to those of coleopterans [11, 12]. Both dipterans and lepidopterans are, along with a three less diverse orders, members of an insect clade called “Mecopterida” (Table 1), and members of these three orders also experienced a strong acceleration of aminoacid substitution rate at least for the ecdysone receptor gene, a major regulator of molting [13]. Taken together, these data suggest that the acceleration of evolutionary rate took place at the stem of Mecopterida. Interestingly, the interaction between the two proteins that make the ecdysone receptor, USP and EcR, was conserved in spite of the acceleration, because it was compensated by the acquisition of a new dimerization surface that stabilized both partners [14]. The fact that the name “Mecopterida” is almost unknown outside entomology circles illustrates the absence of correlation between this strong molecular divergence and any major phenotypic change. In contrast, the two other species-rich insect orders, hymenopterans and coleopterans, have not experienced such a genome-wide acceleration, indicating that the understanding of this process will require more than a simplistic adaptationist scenario.

 In nematodes, similar findings can be made. For example, in the major nematode model species *C. elegans, *one of the most salient genomic features is the presence of some protein families with high numbers of duplications or coding genes. This is the case for the hedgehog-related sterol-binding secreted signaling proteins [15], which are mainly expressed in cuticular cells [16]. It is also the case for the guanylyl cyclases [17], tyrosine kinases [18], seven-transmembrane receptors [19], and nuclear receptors [20], as well as a number of other families that have not been studied specifically in nematodes. The analysis of members of multigene families that duplicated early in metazoan evolution or even before requires detailed phylogenetic investigations of each of these families, which is not always available. Therefore, it is still impossible to provide a comprehensive overview of genome diversification based on single-gene duplications. Moreover, the precise functional meaning of such amplifications remains quite obscure, even if these expansions show readily discernible patterns.

 The functional gene categories most prone to lineage-specific expansions in eukaryotes seem to be structural proteins. This involves enzymes functioning in response to pathogens and environmental stress and includes various components of signaling pathways responsible for specificity, such as ubiquitin ligase subunits and transcription factors [21]. While the duplication pattern of nematode genes is roughly consistent with this notion, lineage-specific variations also exist, especially concerning the spatial distribution of duplicates in the genome. In *C. elegans*, for example, the number of duplicates varies greatly depending on the chromosomal location. The highest concentration of duplicates is found on chromosome V, which reflects tandem amplification in a specifically dynamic chromosomal context and indicates that purely structural factors can also drive the pattern of gene duplication pattern [22]. At least in *C. elegans *and its close relative* C. briggsae, *there is a strong difference with regard to the position along the chromosome, as duplicated genes are more abundant in the chromosomal arms than in the centromeric part [23–25].

 Apart from gene duplications, a major unexpected outcome of nematode genome sequencing is the importance of horizontal gene transfer (HGT), a process that was previously thought to be rare among eukaryotes with sexual reproduction. It turns out that many genes encoding for plant cell-wall modifying proteins were acquired in some nematode lineages many times independently and from various donor organisms [26–28]. Recipients of HGT-acquired cell-wall modifying proteins were plant-parasitic nematodes of the genera *Bursaphelenchus*, *Meloidogyne, Heterodera, Globodera, Pratylenchus, *and* Xiphinema* (for review, see [29]). Additionally, some nonplant parasitic nematodes such as the necromenic species *Pristionchus pacificus* have obtained cellulases from protist-type donors [30].

 A major bottleneck for better understanding gene duplications and other processes such as HGT in their short-term and long-term evolutionary consequences is the lack of precise functional knowledge about the majority of these paralogous genes. This includes the well-studied genetic model system *C. elegans*. Compounded with the absence of functional genetic data for many paralogous genes is that little is known about the population structure and polymorphism rates in *C. elegans*. For example, when positive selection was detected among the *srz* family (the Z family of the serpentine receptor superfamily), where no protein had a precisely known physiological function, there were no additional data that would help to interpret the meaning of this observation [31]. This represents an important challenge for future studies because pure computational detection of candidate gene for positive selection is not sufficient to ascertain that an evolutionary event has a real functional meaning. Indeed, the frequency of adaptative substitutions can sometimes be overestimated due to the interplay of other processes that also influence the frequency of nucleotide substitutions, such as genetic hitchhiking or epistatic effects between nonindependent sites, processes of which vary greatly among lineages [32].

 Biases in codon composition that are taken as molecular signatures for positive selection can also be produced by a specific bias in DNA turnover at that particular part of the genome. Such a mechanism was already suggested 30 years ago under the concept “molecular drive” [33] and got further support by whole-genome studies on the distribution of sites that are predicted to be under positive selection [34, 35]. Advances in population-genetic theory showed the emergence of certain kinds of aminoacid substitutions and protein-protein complexes restricted to taxa with relatively small effective population sizes [36]. Besides this structure-driven effect of gene family amplification, one should also take into account the possibility that the structure of some signaling networks necessitates the retention of duplicates, similar to what occurs in Mecopterida, where many members of the same ecdysone-signaling network have undergone a supplemental acceleration in nucleotide turnover when compared to the rest of the genome [37]. Furthermore, it should be noted that following an original gene duplication, “new” functions that arise subsequently are not really new but represent cases of subfunctionalization and cooption [38]. In addition, it has recently been proposed that there is no definitive proof that orthologous genes are functionally more similar than closely related paralogs [39]. Comparisons of aminoacid substitution patterns between the speciation event that separated insects and chordates and the duplication event at the basis of vertebrate show similar trends, suggesting that speciation is as important as duplication to promote novelty in gene families [40].

 Taken together, genome-wide analyses of species-rich groups of insects and nematodes have identified mechanisms by which genomes can diversify to create novelty in the absence of complete genome duplications. Although both groups show an extraordinary level of genome data and have been studied by the scientific community for more than a century, the functional understanding of genes is often limited. Given space restrictions, we are unable to discuss fully the many hypotheses that have been proposed in association with the limitations of our current understanding, and so we refer the reader to cited literature for more in depth discussions.

## 3. A Case Study: Molecular Novelty in a Conserved Gene Family

**Figure 1:**
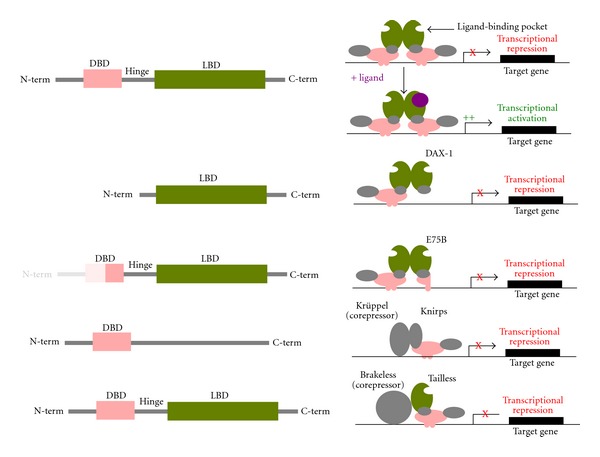
Various levels of functional diversity in a very conserved protein family. The first line shows the canonical structure of a nuclear receptor, comprising a DNA-binding domain (DBD) and a ligand-binding domain, that are structurally well conserved. A canonical receptor represses transcription in absence of a ligand (or it is even not in the nucleus) and activates transcription upon ligand binding. The second line shows a receptor that has lost its DNA-binding domain and that acts also as a transcriptional repressor. The third line shows a receptor that is complete at the gene level, but for which the expression of one isoform starts only at the half of the DNA-binding domain. It acts also as a transcriptional repressor. The fourth line shows a receptor having lost its ligand-binding domain. The last line shows an example of receptor that still has this canonical structure, but that has no known ligand and acts also as a constitutive transcriptional repressor. Whereas knirps and tailless bind to corepressors that are not nuclear receptors, DAX-1 and E75B act as dominant negatives, blocking the activation activity of another nuclear receptor with a canonical structure.

 Gene families have been identified as a major target for the generation of molecular novelty in eukaryotes. One of the gene families with a spectacular duplication rate is the nuclear receptor family. In nematodes, for example, the majority of duplicates arose from the amplification of a single member of the family, named HNF4 (NR2A), up to more than 250 duplicates in *C. elegans* [41]. In general, nuclear receptors are currently defined as ligand-activated transcription factors that can undergo a conformational change in response to the binding of a small molecule [42]. Some studies based on ancestral sequence reconstruction and *in vitro* analysis document up to the level of individual mutations how innovations in ligand-binding ability have arisen [43, 44]. This family is one of the most stable at the metazoan level, its members showing a conserved modular structure comprising a DNA-binding domain and a ligand-binding domain that are well characterized at the structural level [45]. However, even among nuclear receptors, there are some atypical members that can have important functional roles in spite of an altered functional structure (Figure 1). For example, the vertebrate DAX-1 (NR0B) is a receptor that has no DNA-binding domain. It is involved in X-linked adrenal hypoplasia congenita, a developmental disorder of the human adrenal gland, where it acts as a dominant-negative receptor that blocks the activation ability of other nuclear receptors [46]. In *Drosophila*, a similar situation appears during the molting cycle. One of the receptors involved in the regulation of molting, E75 (NR1D), has many isoforms that are expressed at various stages in the molting cycle. The isoform E75B lacks half of its DNA-binding domain, having only one zinc finger instead of two for a canonical DNA-binding domain. Being itself unable to bind DNA, it acts as a transcriptional repressor by blocking the transactivation abilities of its dimerization partner [47]. This example nicely demonstrates that even a single gene can give rise to proteins with different and even antagonistic functions, due to the variability generated by alternative splicing.

 Other members of the nuclear receptor family are devoid of a ligand-binding domain. This is the case for the developmental control gene *knirps* (NR0A1), a *Drosophila* segmentation gene whose expression in the posterior part of the fly embryo is responsible for the presence of abdominal segments 1–7 [48]. It is also involved in head morphogenesis and in tracheal formation later in development. Interestingly, some of its functions in late development are redundant with those of its close paralog *knrl *[49], but the greater intron size of *knrl *relative to* knirps* prevents it from functional complementation duringsegmentation, where transcription time during short mitotic cycles provides a physiological barrier to transcript size [50]. While both genes arose from a duplication event in the cyclorrhaphan diptera, their nonduplicated ortholog in *Tribolium castaneum* also plays a role that is essential for head patterning [51].

**Figure 2:**
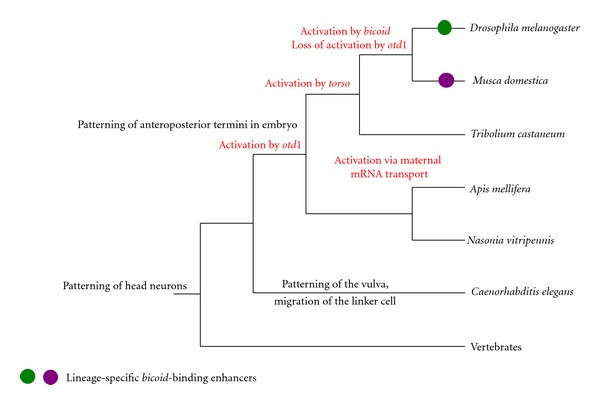
Examples of functional shifts at the level of a single protein. The transcriptional repressor *tailless* is considered to have a conserved function in bilaterians concerning the patterning of anterior neurons. But in addition to that, it has secondarily acquired a number of lineage-specific functions. In *Caenorhabditis elegans*, it contributes to the patterning of the vulva in hermaphrodites and in migration of the linker cell from the male gonad. In holometabolous insects, it participates in the patterning of the anterior and posterior tips of the embryo. Strikingly, even if the expression domain of *tailless* is conserved in holometabolous embryos, this is achieved through highly variable transcriptional pathways (in red on the figure).

Such a patterning role of nuclear receptors during insect segmentation is not restricted to receptors that have lost their ligand-binding domain but can also be observed in the orphan receptor encoded by the *tailless *gene (NR2E2). This protein has a recognizable ligand-binding domain but no known ligand. In *Drosophila*, this transcription factor belongs to the segmentation genes like *knirps*, and it functions in the segmentation gene hierarchy by providing an early subdivision into groups of segments of the embryo by acting as a transcriptional repressor [52]. This function seems to be conserved among holometabolous insects [53]. However, the most conserved part of* tailless *function seems to be its role in the specification of the anterior nervous system (Figure 2). In *Drosophila*, *tailless* controls the formation of the protocerebral neuroblasts and acts in eye formation [54]. In mammals, it is involved in brain and visual system development, as well as in neural stem-cell renewal at the adult stage [55]. In *C. elegans*, the* tailless* ortholog, named *nhr-67*, is expressed in six head neurons, but is also involved in other processes, such as the patterning of the vulva [56] and the control of the migration of the linker cell in the male gonad [57].

 All these examples from nuclear receptors that do not act as ligand-activated transcription factors show that even in a family that is very well conserved at the structural level, there can be many functional variations and novelty. These unusual cases are involved in the control of important biological processes, providing a powerful first glance at the complexity of eukaryotic genomes. One should be aware, however, that such studies can lead only to partial conclusions, which need to be completed by more precise functional investigations. The genes described above, like any other gene in eukaryotic genomes, might have acquired novel but simple protein domains, that are not easily detectable by bioinformatic means. For example, the origin of four amino acid SH3-binding domains in an otherwise conserved LIN-18/Ryk/derailed receptor in WNT signaling has allowed new wiring in the signaling pathway leading to vulva formation in the nematode *Pristionchus pacificus* but which is not present in *C. elegans* [58]. Such domains would go unnoticed without functional studies by unbiased genetic approaches.

## 4. * cis*-Regulatory Novelties in the Promoter of a Gene with Conserved Expression Pattern

 In the recent years, there has been an ongoing debate about the contribution of *cis*-regulatory elements versus protein-coding regions in evolutionary innovation [59, 60]. The arguments are extensively reviewed in detail elsewhere [61], so we will concentrate on the complementary side of the problem, the fact that high promoter turnover and changes in transcriptional regulation are compatible with a conserved gene expression pattern.

 The evolution of the promoter of the *tailless* gene is especially well studied in holometabolous insects (Figure 2). The expression of the *tailless *gene in *Drosophila melanogaster* is regulated by a complex set of transcription factors, the most important being *bicoid* [62] and two other genes that are in the downstream *torso* signaling pathway [63]. The promoter of another dipteran fly, *Musca domestica*, contains binding sites for all these transcription factors in similar numbers, although the binding sites are organized in a different order. The expression of *tailless* is highly similar in the two flies at the blastodermal stage [64], the only subtle difference being the split of the expression pattern in the anterior cap from the *Musca* embryo, which does not occur in *Drosophila. *Additionally, the promoter of *Musca* is able to drive a *Drosophila*-like expression pattern of a reporter gene when inserted in *Drosophila* embryos [65]. These observations completed by estimations about the mutation rates in *Drosophila* gene promoters suggest that the promoter region was fully renewed during the 100 million years following the divergence of *Drosophila* and *Musca *[65]. It has therefore been argued that the regulation by the same transcription factors was maintained by constant loss and *de novo* acquisitions of similar promoter elements [65].

 In the flour beetle *Tribolium castaneum*, the expression pattern of *tailless* during embryogenesis is similar to that in flies, and the activation by the *torso* pathway is conserved as well. In contrast, transcriptional control by *bicoid* is not possible, because *bicoid* is specific to flies [66]. In contrast to flies and beetles, hymenopterans represent again a different case. In the parasitic wasp *Nasonia vitripennis*, in which components of the *torso* pathway are missing, the expression of *tailless* is activated by *orthodenticle-1* [67]. In the honeybee *Apis mellifera*, anterior expression of *tailless* also depends on *orthodenticle-1*, but its posterior expression is due to maternal RNA [67]. A contribution of *orthodenticle-1 *to* tailless *expression also in *T. castaneum *is likely, given the fact that *orthodenticle-1* is a proposed substitute for* bicoid *in this insect [68], but direct binding from *orthodenticle-1* to the *tailless* promoter is yet to be reported. In spite of this remaining question, the comparison of the regulatory mechanisms for the *tailless* expression shows that there are already four slightly different ways that are known to maintain this pattern in holometabolous insects.

 The *tailless* case is particularly well documented in terms of species sampling. Yet there are many other examples of high promoter turnover in genes whose expression is conserved (reviewed in [69]). This illustrates the notion of developmental system drift, describing the fact that many changes in developmental pathways occur during evolution without phenotypic effect and thus are more likely to be the result of contingent historical events than the response to selection pressure [70].

## 5. Conclusion

In his autobiography, Darwin [71] wrote the following about the reasons that pushed him to write two extensive monographies about cirripedes: ‘‘*When on the coast of Chile, I found a most curious form, which burrowed into the shells of Concholepas, and which differed so much from all other Cirripedes that I had to form a new sub-order for its sole reception. […] To understand the structure of my new Cirripede, I had to examine and dissect many of the common forms: and this gradually led me on to take up the whole group.'*' This illustrates perfectly well what lies on the agenda of today's evolutionary biologists. What has changed since Darwin's time is that now we have the tools required to describe natural variation from the molecular level to the ecological one. It follows that, for a given node of a phylogenetic tree, variation and repartition of characters can and need to be addressed at all these levels. What we have to understand is the connection between the various layers of biological complexity, combining and integrating the results of laboratory and fieldwork approaches [6].

 We argue that the partial data on genomic variation are already sufficient to indicate that no specific attribute of a given molecular structure can indicate *a priori* more potentials than others to contribute to novelty at a higher phenotypic level. Uncoupling and buffering of natural variation at various integration scales has been clearly demonstrated, implying that the number of molecular events that can be directly correlated with a phenotypic change at the organismal level is probably very low, and that they are the results of exceptional contingency and structural constraints [72]. The possibility to detect *de novo* such interesting changes in nonmodel organisms is thus also probably very low, and it decreases quickly with an increase of the phylogenetic distance. Additionally, one should not forget that to really understand the link between genetic variation and phenotypic diversity, it is necessary to be able to explain cases where a molecular change triggers novelty, and those where phenotypic traits are maintained in spite of molecular innovations in the genes that specify them.

## Figures and Tables

**Table 1:** Decoupling between species number and genome acceleration rates in holometabolous insects.

Order	Approximate number of described species
Diptera	150 000
Mecoptera	600
Siphonaptera	1750
Trichoptera	7000
Lepidoptera	120 000

Total mecopterida	279 350 species

Coleoptera	350 000
Strepsiptera	600
Hymenoptera	115 000
Neuroptera	6000
Raphidioptera	210
Megaloptera	300

Total nonmecopterida	472 110 species
